# Coal dust-stimulated macrophage-derived extracellular vesicles promote thyroid cancer cell phenotypes involving miR-205-5p, CAMK1G, and NF-κB-associated signaling

**DOI:** 10.3389/fimmu.2026.1856770

**Published:** 2026-10-14

**Authors:** Feng Zhao, Tianqing Ma, Chi Zhang, Hongzhen Zhang

**Affiliations:** 1 The First Affiliated Hospital of Anhui University of Science and Technology, Huainan, China; 2 School of Medicine, Anhui University of Science & Technology, Huainan, China; 3 Anhui University of Science and Technology, Huainan, China

**Keywords:** CAMK1G, coal dust, macrophage-derived extracellular vesicles, miR-205-5p, NF-κB signaling, occupational Exposure, thyroid cancer

## Abstract

**Background:**

Occupational coal dust exposure causes persistent pulmonary inflammation, but the mechanisms by which it may influence thyroid cancer progression remain unclear. Macrophage-derived extracellular vesicles (EVs) may participate in intercellular communication under particulate stress.

**Objective:**

This study investigated whether EVs released by coal dust-stimulated macrophages alter thyroid cancer cell behavior and examined the involvement of miR-205-5p, CAMK1G, and NF-κB-associated signaling.

**Methods:**

Coal dust was physicochemically characterized before use. EVs were isolated from PMA-differentiated THP-1 macrophages following coal dust exposure and characterized by transmission electron microscopy, nanoparticle tracking analysis, and Western blotting. Small-RNA sequencing, bulk RNA sequencing, TCGA-THCA analysis, and the GSE274674 single-cell dataset were integrated with RT-qPCR, nuclear–cytoplasmic fractionation, dual-luciferase reporter assays, and genetic rescue experiments. Coal dust-exposed C57BL/6J mice and PBMC-humanized NSFG mice bearing TPC-1 xenografts were used for *in vivo* evaluation.

**Results:**

PKH26-labeled EV-associated material was detected in TPC-1 cells, and EV preparations increased cell viability, clonogenic growth, migration, and invasion while reducing apoptosis. miR-205-5p was enriched in these EVs and accumulated predominantly in the nuclear fraction of recipient cells. Inhibition of miR-205-5p attenuated the EV-associated malignant phenotypes. Integrated transcriptomic analyses prioritized CAMK1G and NF-κB-related signaling. EV treatment increased endogenous CAMK1G expression and the phosphorylation of p65 and IκBα. CAMK1G knockdown reduced these changes, whereas CAMK1G re-expression partially restored the effects suppressed by miR-205-5p inhibition. The reporter assay showed sequence-dependent repression of the isolated CAMK1G 3′-UTR construct, whereas endogenous-expression and rescue experiments supported a positive functional association; therefore, these findings are not interpreted as a simple direct positive regulatory relationship. In mice, coal dust exposure was associated with pulmonary abnormalities, altered serum FT4 and TPO-Ab levels, increased thyroid CAMK1G expression, and enhanced pulmonary CD206 immunoreactivity. In PBMC-humanized mice, coal dust exposure or experimentally administered macrophage-derived EVs increased xenograft growth, whereas CAMK1G knockdown attenuated tumor burden.

**Conclusion:**

These findings support a functional association between coal dust-stimulated macrophage-derived EV signaling and thyroid cancer progression involving miR-205-5p, CAMK1G, and NF-κB-associated activity. However, direct physiological trafficking of endogenous lung-derived EVs to thyroid or tumor tissue was not demonstrated.

## 1 Introduction

Coal workers’ pneumoconiosis (CWP) is a chronic occupational lung disease caused by long-term inhalation and deposition of coal mine dust. Despite improvements in occupational protection and dust-control strategies, CWP remains a significant occupational health challenge and may continue to progress even after exposure cessation. Previous studies have demonstrated the persistent and progressive burden of CWP, including progression after exposure cessation and substantial disease-associated mortality (1–3). In addition to pulmonary injury, coal dust exposure has been associated with systemic metabolic and immunological alterations (4, 5). However, whether chronic coal dust exposure influences diseases beyond the respiratory system and the molecular mechanisms underlying these potential systemic effects remain incompletely understood.

Macrophages represent one of the first lines of cellular defense against inhaled particulate matter in the lung. Persistent stimulation by coal dust particles can alter macrophage activation states, inflammatory responses, and secretory profiles. Extracellular vesicles (EVs) have emerged as important mediators of intercellular communication by transporting proteins, lipids, and regulatory RNAs between cells. EV-associated miR-205-5p has been implicated in angiogenesis, cell migration, toxicant-associated immune injury, and macrophage-related inflammatory responses (6–8). Therefore, macrophage-derived EVs represent a potential mechanism through which particle-induced macrophage responses may influence distant cellular environments.

MicroRNAs (miRNAs) are small non-coding RNAs that regulate gene expression through sequence-dependent interactions with target transcripts and play essential roles in immune regulation, inflammation, and tumor progression (9). Among these molecules, miR-205-5p has attracted increasing attention because of its context-dependent involvement in cancer biology (6, 10, 11). Previous studies have shown that EV-associated miR-205-5p participates in angiogenic regulation and cellular migration (6), contributes to toxicant-induced immune injury through modulation of macrophage polarization (7), and regulates inflammatory lung injury through effects on macrophage function (8). These findings indicate that miR-205-5p may serve as an important regulatory molecule transported through EV-mediated intercellular communication.

Occupational coal dust exposure has also been associated with thyroid abnormalities. An epidemiological study in coal miners reported an association between occupational characteristics and nodular thyroid disease, suggesting a possible relationship between occupational exposure and thyroid dysfunction (12). Our previous study further implicated pulmonary EV-associated miR-205-5p in CWP-related nodular thyroid disease and suggested that EV-mediated miRNA communication may contribute to thyroid-related pathological changes (13). However, the specific cellular source of these EVs, their effects on thyroid cancer cells, and the downstream molecular mechanisms remain unclear.

Thyroid cancer progression is regulated by complex interactions between genetic alterations, inflammatory signaling, and tumor microenvironmental factors. Calcium/calmodulin-dependent protein kinase 1G (CAMK1G), also known as CLICK-III/CaMKIγ, is a membrane-associated calcium/calmodulin-dependent kinase involved in calcium-responsive signaling processes (14). Although CAMK1G has been characterized in neuronal and calcium-dependent signaling contexts, its function in thyroid cancer remains largely unexplored. The nuclear factor-κB (NF-κB) pathway is a central inflammatory signaling pathway involved in tumor-cell proliferation, migration, invasion, and resistance to apoptosis. Whether CAMK1G participates in EV-associated thyroid cancer progression through modulation of NF-κB signaling remains unknown.

In this study, we investigated whether EVs derived from coal dust-stimulated macrophages influence thyroid cancer cell behavior and explored the potential involvement of miR-205-5p, CAMK1G, and NF-κB-associated signaling. Coal dust particles were first characterized using physicochemical approaches. EVs isolated from coal dust-treated macrophages were evaluated by transmission electron microscopy, nanoparticle tracking analysis, and EV marker detection. By integrating small-RNA sequencing, transcriptomic analysis, public thyroid cancer datasets, molecular intervention, and rescue experiments, we identified CAMK1G as a candidate mediator associated with EV-induced cellular responses. Furthermore, coal dust exposure models and PBMC-humanized xenograft models were used to evaluate the functional relevance of this regulatory network *in vivo*. Because endogenous lung-derived macrophage EVs were not directly traced to thyroid or tumor tissues, this study provides functional evidence supporting an EV-associated regulatory mechanism rather than definitive proof of physiological lung-to-thyroid EV trafficking.

## 2 Materials and methods

### 2.1 Ethics statement

All animal procedures were reviewed and approved by the Institutional Ethics Committee of the First Affiliated Hospital of Anhui University of Science and Technology (approval no. GZ2025-07) and were conducted in accordance with institutional guidelines for the care and use of laboratory animals. Commercial human peripheral blood mononuclear cells (PBMCs) were obtained in a de-identified form from Nuohang Biotechnology Research Institute (Xuzhou, China). No participants were prospectively recruited, and no newly collected human specimens or identifiable clinical data were used in this study. Analyses of The Cancer Genome Atlas (TCGA) and Gene Expression Omnibus (GEO) datasets were based exclusively on publicly available, de-identified data.

### 2.2 Cell culture and macrophage differentiation

Human monocytic cell lines THP-1 and U937 and human thyroid cancer cell lines TPC-1 and ACT-1 were used. THP-1 and U937 cells were maintained in RPMI-1640 medium, whereas TPC-1 and ACT-1 cells were cultured in high-glucose Dulbecco’s modified Eagle’s medium. All media were supplemented with 10% fetal bovine serum and 1% penicillin–streptomycin, and cells were maintained at 37 °C in a humidified atmosphere containing 5% CO_2_. THP-1 cells were treated with 100 ng/mL phorbol 12-myristate 13-acetate for 48 h to induce differentiation into adherent macrophage-like cells. The cells were subsequently washed with phosphate-buffered saline (PBS) and allowed to recover in fresh complete medium for 24 h before coal dust exposure. For extracellular-vesicle collection, the culture medium was replaced with medium containing 10% exosome-depleted fetal bovine serum.

### 2.3 Coal dust preparation and characterization

Bituminous coal dust was collected from an underground fully mechanized working face of a state-owned coal mine in Huainan, Anhui Province, China. The samples were naturally dried, passed through a 200-mesh sieve, and ground for 10 min. For animal experiments, coal dust was suspended in sterile saline at 240 μg/mL, ultrasonically dispersed for 30 min, and sterilized at 121 °C for 20 min. For cell experiments, the suspension was freshly dispersed by ultrasonication and added to the culture medium at a final concentration of 100 μg/mL. The *in vitro* concentration of 100 μg/mL was selected on the basis of preliminary dose-response experiments, in which this concentration produced a reproducible macrophage response without excessive loss of cell viability. The animal exposure regimen was reported exactly as performed and was applied consistently across the *in vivo* experiments; because a separate *in vivo* dose-ranging study was not conducted, it is not presented as an optimized or human-equivalent exposure dose. Coal dust morphology was examined by scanning electron microscopy at low and high magnifications. Elemental composition and spatial distribution were evaluated using energy-dispersive X-ray spectroscopy. Chemical functional groups were characterized by Fourier-transform infrared spectroscopy, and the hydrodynamic particle-size distribution was measured by dynamic light scattering using three independently prepared suspensions.

### 2.4 Screening of macrophage–thyroid cancer cell combinations

To screen macrophage–thyroid cancer cell combinations, EV preparations derived from coal dust-exposed THP-1 or U937 macrophage-like cells were separately applied to TPC-1 or ACT-1 thyroid cancer cells, generating four donor/recipient combinations (THP-1/TPC-1, THP-1/ACT-1, U937/TPC-1, and U937/ACT-1). No direct physical co-culture of macrophages and thyroid cancer cells was performed in this screening step. Thyroid cancer cell viability was evaluated using the Cell Counting Kit-8 assay at the indicated time points. After addition of the CCK-8 reagent and incubation according to the manufacturer’s protocol, absorbance was measured at 450 nm. The THP-1/TPC-1 combination, which showed the most pronounced proliferative response, was selected for subsequent experiments.

### 2.5 Isolation and characterization of macrophage-derived extracellular vesicles

Differentiated THP-1 cells were exposed to 100 μg/mL coal dust for 24 h. Untreated cells served as controls. Conditioned media were collected and centrifuged at 300 × g for 10 min and 2,000 × g for 20 min to remove cells and large debris. The supernatants were passed through 0.22-μm filters and concentrated using 100-kDa molecular-weight-cutoff centrifugal filters. Extracellular-vesicle-enriched fractions were then isolated using Total Exosome Isolation reagent for cell culture media (Thermo Fisher Scientific, Waltham, MA, USA; catalog no. 4478359) according to the manufacturer’s instructions. Three independent biological samples were prepared for each condition.

Extracellular-vesicle-enriched preparations isolated from untreated macrophages were designated Ctrl-EV, whereas those isolated from coal dust-exposed macrophages were designated CD-EV. Throughout the revised manuscript, EV terminology is used for the study-generated preparations because their endosomal biogenesis was not directly established. The commercial name “Total Exosome Isolation reagent” is retained only as the manufacturer’s product name. A small number of original figure panels contain legacy labels such as “Exo” or “EXO”; these labels refer to the same EV-enriched preparations and should not be interpreted as evidence of exosome-specific biogenesis. Vesicle protein concentrations were determined using a bicinchoninic acid assay, and TPC-1 cells were treated with EV preparations at a final protein concentration of 30 μg/mL unless otherwise stated. EV morphology was examined using a Hitachi HT7700 transmission electron microscope. Particle size and concentration were determined by nanoparticle tracking analysis. EV enrichment was further assessed by Western blotting for CD9, CD81, and TSG101, with Calnexin used as a negative cellular marker.

### 2.6 PKH26 labeling and cellular uptake

Extracellular vesicles were labeled with PKH26 fluorescent dye. PKH26 and Diluent C were mixed at a ratio of 1:9, and the vesicles were incubated with the staining solution for 10 min at room temperature in the dark. Unbound dye was removed by ultracentrifugation at 120,000 × g for 90 min. PKH26-labeled vesicles were then added to TPC-1 cells and incubated for 12 h. The cells were washed three times with PBS, fixed, and stained with fluorescent phalloidin and Hoechst 33342 to visualize F-actin and nuclei, respectively. Vesicle internalization was examined using a laser-scanning confocal microscope (UltraVIEW VoX/Olympus IX81).

### 2.7 Small-RNA sequencing

Small RNA was extracted from Ctrl-EV and CD-EV, with three independent biological replicates in each group. After quality assessment, small-RNA libraries were prepared and sequenced on an Illumina NovaSeq 6000 platform by Novogene Co., Ltd. Adapter sequences and low-quality reads were removed, and clean reads were mapped to the human reference genome. Mature miRNAs were annotated against the miRBase database. Differential miRNA expression was analyzed after normalization of read counts. Differentially expressed miRNAs were defined by an absolute log_2_ fold change greater than 1 and a Benjamini–Hochberg-adjusted P value below 0.05. miR-205-5p was selected for further validation based on its differential abundance and subsequent RT-qPCR results. The original raw sequencing reads from this in-house small-RNA sequencing experiment have been deposited in the NCBI Sequence Read Archive (SRA) under BioProject accession PRJNA1510000. Processed count data and associated sample metadata are available from the corresponding author upon reasonable request. This in-house dataset is distinct from the public single-cell RNA-sequencing dataset GSE274674 described in Section 2.10.

### 2.8 RNA extraction, nuclear–cytoplasmic fractionation and RT-qPCR

Total RNA was extracted from cells, tissues or extracellular-vesicle preparations using TRIzol reagent. RNA concentration and purity were determined spectrophotometrically, and samples with an A_260_/_A280_ ratio between 1.8 and 2.0 were used for subsequent analyses. Nuclear and cytoplasmic RNA fractions were isolated from TPC-1 cells following incubation with CD-EV. The abundance of miR-205-5p in each fraction was determined by RT-qPCR. Complementary DNA was synthesized using miRNA- or mRNA-specific reverse-transcription reagents, as appropriate. RT-qPCR was performed in a 20-μL reaction containing cDNA, gene-specific primers and SYBR Green Master Mix. The amplification programme consisted of initial denaturation at 95 °C for 10 min, followed by 40 cycles at 95 °C for 15 s and 60 °C for 30 s. Melt-curve analysis was performed to confirm amplification specificity. Relative expression was calculated using the 2^-^ΔΔCt method. U6 and GAPDH were used as internal controls for miRNA and mRNA, respectively. All reactions were performed in triplicate.

### 2.9 Bulk RNA sequencing and enrichment analysis

TPC-1 cells were treated with Ctrl-EV or CD-EV, with three independent biological replicates per group. Total RNA was extracted and stored at −80 °C until sequencing. RNA integrity was assessed using an Agilent Bioanalyzer, and samples with an RNA integrity number of at least 7.0 were used for library preparation. Libraries were sequenced on an Illumina NovaSeq 6000 platform by Novogene Co., Ltd., with at least 30 million clean reads generated per sample. After quality control, clean reads were aligned to the GRCh38 human reference genome, and gene-level read counts were generated. Downstream analyses were performed in R version 4.3.1. Differential expression analysis was performed using DESeq2 version 1.42.0. Genes with an absolute log_2_ fold change greater than 1 and an adjusted P value below 0.05 were considered differentially expressed. Gene Ontology and Kyoto Encyclopedia of Genes and Genomes enrichment analyses were performed using clusterProfiler version 4.10.0. P values were corrected using the Benjamini–Hochberg method, and terms with adjusted *P* values below 0.05 were considered significantly enriched. CAMK1G was prioritized for experimental validation based on the combined transcriptomic, pathway-enrichment, and functional analyses. The original raw sequencing reads from this in-house bulk RNA-sequencing experiment have been deposited in the NCBI SRA under BioProject accession PRJNA1510000. Processed count data and associated sample metadata are available from the corresponding author upon reasonable request. This in-house dataset is distinct from the public single-cell RNA-sequencing dataset GSE274674 described in Section 2.10.

### 2.10 Analysis of TCGA and single-cell RNA-sequencing data

RNA-sequencing and clinical data for the TCGA Thyroid Carcinoma project (TCGA-THCA) were obtained from the NCI Genomic Data Commons. The expression analysis included 505 primary thyroid carcinoma tissues and 59 normal thyroid tissues. Expression values were converted to log_2_(TPM + 1), and CAMK1G expression was compared between tumor and normal tissues using the Wilcoxon rank-sum test. For exploratory overall-survival analysis, patients with complete survival information were divided into high- and low-CAMK1G groups using the median CAMK1G expression level as the cutoff, and Kaplan–Meier curves were compared using the log-rank test. No Cox regression result is used in the revised manuscript. Because of the limited number of deaths in TCGA-THCA, the survival analysis was treated as exploratory and was not used to support prognostic claims. The single-cell RNA-sequencing dataset GSE274674 was obtained from GEO and comprised 15 papillary thyroid carcinoma-related samples, including primary tumors, metastatic lymph nodes, and adjacent normal tissues. Single-cell analyses were performed in R version 4.3.1 using Seurat version 5.0.3 for quality control, normalization, integration, dimensionality reduction, clustering, and cell-type annotation, and AUCell version 1.28.0 for CAMK1G-associated gene-set activity scoring. Thyroid tumor cells were divided into high- and low-CAMK1G groups, followed by GO and KEGG enrichment analyses using clusterProfiler version 4.10.0. Multiple-testing correction was performed using the Benjamini–Hochberg method. The software versions reported here correspond to the analysis environment used for the final analyses. GSE274674 applies only to the public single-cell dataset used for secondary analysis; it is not an accession for the small-RNA or bulk RNA-sequencing datasets generated in the present study.

### 2.11 Dual-luciferase reporter assay

The predicted miR-205-5p-responsive region within the CAMK1G 3′ untranslated region was cloned into a firefly luciferase reporter vector to generate the wild-type construct. A corresponding mutant construct was generated by replacing the predicted miR-205-5p-responsive nucleotides. TPC-1 cells were co-transfected with the wild-type or mutant reporter plasmid and miR-205-5p mimics or the corresponding negative-control oligonucleotide using Lipofectamine 3000 (Invitrogen, USA). Luciferase activities were measured 48 h after transfection using a dual-luciferase reporter assay system. Firefly luciferase activity was normalized to Renilla luciferase activity. Each experiment was independently repeated three times.

### 2.12 Cell transfection and gene intervention

TPC-1 cells were transfected at 60–70% confluence. Anti-miR-205-5p or the corresponding negative-control oligonucleotide was used at a final concentration of 30 nM. CAMK1G expression was reduced using CAMK1G-targeting short-hairpin RNA and increased using a CAMK1G overexpression plasmid. Corresponding non-targeting shRNA and empty-vector controls were included. All transfections were performed using Lipofectamine 3000 according to the manufacturer’s instructions. Stable cells were selected using the appropriate antibiotic and validated by RT-qPCR and Western blotting. Rescue experiments were conducted by restoring CAMK1G expression in cells treated with anti-miR-205-5p.

### 2.13 Western blotting

Cells, tissues or extracellular-vesicle preparations were lysed in RIPA buffer containing protease and phosphatase inhibitors. Lysates were centrifuged at 12,000 rpm for 10 min at 4 °C, and protein concentrations were measured using a bicinchoninic acid assay. Equal amounts of protein were separated by SDS–PAGE and transferred onto polyvinylidene difluoride membranes at 300 mA for 40 min. Membranes were blocked with 5% non-fat milk or bovine serum albumin for 1 h and incubated overnight at 4 °C with primary antibodies against CAMK1G, phosphorylated p65, total p65, phosphorylated IκBα, total IκBα, BAX, caspase-3, CD9, CD81, TSG101, Calnexin, β-actin or GAPDH. After incubation with horseradish peroxidase-conjugated secondary antibodies, protein bands were visualized using enhanced chemiluminescence and quantified using ImageJ. Phosphorylated proteins were normalized to the corresponding total proteins, and other target proteins were normalized to β-actin or GAPDH.

### 2.14 Cell functional assays

For colony-formation assays, TPC-1 cells were seeded in 6-well plates at 500 cells per well and cultured for 10–14 days. Colonies were fixed with 4% paraformaldehyde, stained with crystal violet and counted. For wound-healing assays, cells were cultured to approximately 90% confluence, and a linear scratch was generated using a sterile pipette tip. Detached cells were removed by PBS washing, and images were obtained at 0 and 24 h. Wound closure was quantified from the change in wound area. For Transwell migration assays, 1 × 10^5^ cells suspended in serum-free medium were added to the upper chamber. For invasion assays, the upper chamber was precoated with Matrigel. Medium containing fetal bovine serum was added to the lower chamber. After 24–48 h, cells on the lower surface were fixed, stained with crystal violet and counted in five randomly selected fields. For apoptosis analysis, cells were collected and resuspended in 100 μL of binding buffer. Annexin V-FITC and propidium iodide were added, and the samples were incubated for 15 min at room temperature in the dark. Apoptosis was measured by flow cytometry, with total apoptosis defined as the sum of early and late apoptotic populations.

### 2.15 Coal dust exposure in C57BL/6J mice

A total of 66 male C57BL/6J mice, aged 6–8 weeks and weighing 20–22 g, were used to assess short-term thyroid-function changes and longer-term pulmonary and thyroid responses to coal dust exposure. Male mice were selected because the occupational population motivating this study—coal miners—is predominantly male. This sex-restricted design was intended to reflect the principal exposed workforce rather than to establish sex-independent effects; female mice were not included, and potential sex-specific responses were therefore not assessed. For the short-term time-course experiment, 60 mice were randomly assigned to a coal dust exposure group or saline control group (30 mice per group). Mice in the exposure group received 125 μL of coal dust suspension at 240 μg/mL by intratracheal instillation once daily, whereas control mice received an equivalent volume of sterile saline through the same route. Five mice per group were euthanized at 0.5, 1, 2, 4, 7, and 14 days after the start of exposure, and serum was collected for FT4 and TPO-Ab measurement. Thyroid tissues collected from the day-7 and day-14 cohorts were used for the 1- and 2-week CAMK1G immunohistochemical analyses, respectively. An additional 4-week cohort of six mice (n = 3 per group) received the same exposure or control regimen and was maintained through day 28. Micro-computed tomography was performed on day 28, after which thyroid tissues from this cohort were collected for the 4-week CAMK1G immunohistochemical analysis. This design separates the short-term serum time course from the longer 28-day pulmonary imaging and thyroid-histology assessment. A formal blinding procedure was not prospectively documented for these animal experiments.

### 2.16 PBMC-humanized mice and subcutaneous xenografts

NSFG mice aged 4–6 weeks received 1 × 10^7^ human PBMCs per mouse by tail-vein injection. Peripheral blood was collected at days 7, 14, and 38 after transplantation. Human immune-cell engraftment was assessed by flow-cytometric detection of human CD45 and mouse CD45. Successful humanization was defined as human CD45-positive cells accounting for at least 20% of peripheral-blood CD45-positive leukocytes. Humanization was confirmed on day 38 after PBMC transplantation, and only mice meeting the prespecified hCD45 criterion proceeded to tumor implantation. On day 38, these mice were injected subcutaneously with 1 × 10^6^ stable sh-NC or sh-CAMK1G TPC-1 cells and were randomly assigned to six groups: sh-NC plus vehicle, sh-NC plus coal dust, sh-NC plus CD-EV, sh-CAMK1G plus vehicle, sh-CAMK1G plus coal dust, and sh-CAMK1G plus CD-EV. Each group contained four mice. Coal dust was administered using the exposure protocol described above. CD-EV were administered through the tail vein at 10 μg per mouse once every 3 days until the study endpoint. Tumor dimensions and body weight were recorded regularly. Tumor volume was calculated as length × width²/2. At day 15 after tumor implantation, mice were euthanized by controlled CO_2_ inhalation, and tumors were excised, photographed, and weighed. Humane endpoints included a loss of more than 20% of baseline body weight, severe respiratory distress, complete restriction of normal activity, tumor ulceration, or other signs of substantial pain or distress. A formal blinding procedure was not prospectively documented. The complete animal study design is summarized in **Table 1**.

**Table 1:** Summary of the animal study design.

Model/purpose	Groups	n	Intervention	Timing	Primary assessments
C57BL/6J short-term thyroid-function time course	Coal dust vs saline	30/group; 5/group/time point	125 μL coal dust suspension (240 μg/mL) or saline by intratracheal instillation once daily	0.5, 1, 2, 4, 7, and 14 d	Serum FT4 and TPO-Ab; thyroid CAMK1G IHC at day 7 (1 week) and day 14 (2 weeks)
C57BL/6J 4-week pulmonary/thyroid cohort	Coal dust vs saline	3/group	Same coal dust or saline regimen	Day 28	Lung micro-CT; thyroid CAMK1G IHC (4 weeks)
PBMC-humanized NSFG subcutaneous xenograft model	sh-NC + vehicle; sh-NC + coal dust; sh-NC + CD-EV; sh-CAMK1G + vehicle; sh-CAMK1G + coal dust; sh-CAMK1G + CD-EV	4/group	1 × 10^7^ PBMCs i.v.; humanization confirmed on day 38; 1 × 10^6^ TPC-1 cells s.c. on day 38; coal dust as above; CD-EV 10 μg i.v. every 3 d	hCD45/mCD45 at days 7, 14, and 38 after PBMC transfer; tumor implantation on day 38 after PBMC transfer; tumor endpoint 15 d after implantation	Humanization by flow cytometry; body weight; tumor volume/weight; tumor CAMK1G IHC; lung CD206 IHC

### 2.17 ELISA and immunohistochemistry

Serum free thyroxine and thyroid peroxidase antibody concentrations were measured using commercial mouse ELISA kits according to the manufacturers’ instructions. Concentrations were calculated from standard curves. Thyroid, tumor and lung tissues were fixed in 10% neutral-buffered formalin, embedded in paraffin and sectioned. Following deparaffinization, rehydration and antigen retrieval, the sections were incubated overnight at 4 °C with primary antibodies against CAMK1G or CD206. Staining was developed using 3,3′-diaminobenzidine and counterstained with hematoxylin. Staining was evaluated using a semiquantitative immunoreactivity score calculated by multiplying the positive-cell proportion score by the staining-intensity score. Positive-cell proportions were scored as 0, 0%; 1, 1–25%; 2, 26–50%; 3, 51–75%; and 4, 76–100%. Staining intensity was scored as 0, absent; 1, weak; 2, moderate; and 3, strong. The resulting score ranged from 0 to 12. At least five non-overlapping fields were evaluated per section.

### 2.18 Statistical analysis

Statistical analyses were performed using IBM SPSS Statistics version 26.0 and GraphPad Prism version 8.0. Unless otherwise specified, all cell experiments were independently repeated at least three times. Technical replicates and multiple fields obtained from the same biological sample were averaged before analysis. Continuous data are presented as the mean ± standard deviation. Normality was assessed using the Shapiro–Wilk test, and homogeneity of variance was evaluated using Levene’s test. Comparisons between two groups were performed using an unpaired two-tailed Student’s t-test or Mann–Whitney U test, as appropriate. Comparisons among more than two groups were performed using one-way analysis of variance followed by Tukey’s test or the Kruskal–Wallis test followed by Dunn’s test. Experiments involving treatment and time were analyzed using two-way analysis of variance followed by Šídák’s multiple-comparison test. Longitudinal measurements from the same animals were analyzed using repeated-measures analysis of variance. For sequencing analyses, *P* values were corrected using the Benjamini–Hochberg false-discovery-rate method. Kaplan–Meier survival curves were compared using the log-rank test. All statistical tests were two-sided, and *P* < 0.05 was considered statistically significant. Significance was denoted as *P < 0.05, **P < 0.01 and ***P < 0.001. Exact biological sample sizes and statistical tests are provided in the corresponding figure legends.

## 3 Results

### 3.1 Physicochemical characterization of coal dust

Scanning electron microscopy revealed that the coal dust consisted of heterogeneous particles with irregular, angular, and fragmented morphologies. At low magnification, the particles exhibited a broad range of sizes, whereas high-magnification imaging showed rough surfaces, sharp edges, fracture planes, and smaller mineral-like deposits attached to larger particles (**Figures 1A, B**). Energy-dispersive X-ray spectroscopy showed that carbon and oxygen were broadly distributed throughout the particles, together with lower levels of nitrogen, aluminum, silicon, sulfur, and other mineral-associated elements (**Figure 1C**). The corresponding energy spectrum further confirmed that carbon was the predominant component, accompanied by oxygen and trace amounts of several inorganic elements, including aluminum, silicon, calcium, titanium, iron, nickel, selenium, and zirconium (**Figure 1D**). Fourier-transform infrared spectroscopy identified characteristic absorption bands attributable to hydroxyl, carbonyl, N–H, Si–O–Si, and Fe–C-associated vibrations, supporting the presence of both carbonaceous and inorganic mineral constituents (**Figure 1E**). Dynamic light scattering showed similar, predominantly unimodal particle-size distributions among three independently prepared suspensions, with the major peaks located within the submicron range (**Figure 1F**). Together, these results confirmed that the exposure material was compositionally complex coal dust rather than purified SiO_2_.

**Figure 1:**
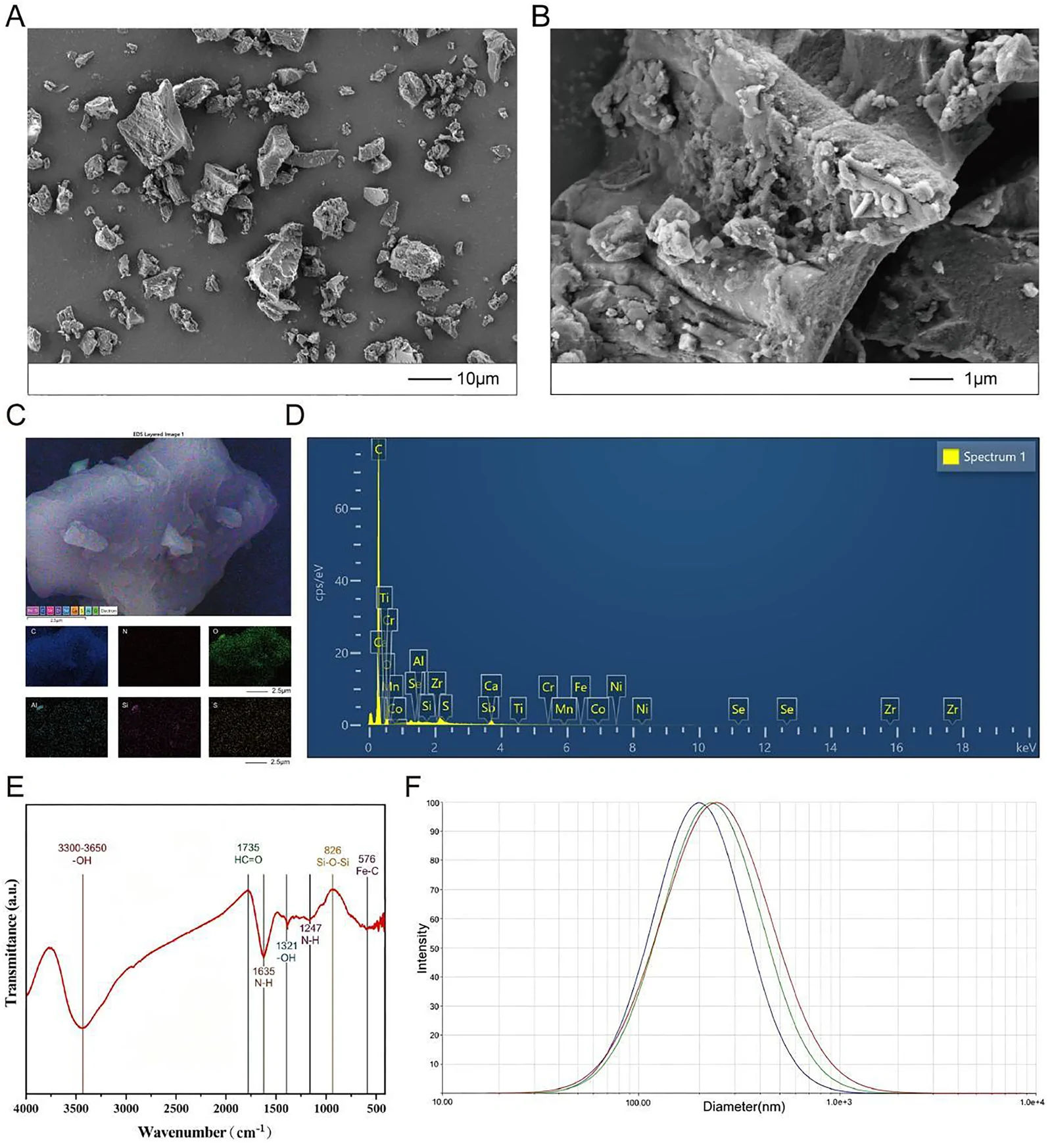
Physicochemical characterization of the coal dust used in this study. (**A, B**) Representative scanning electron microscopy images showing the heterogeneous morphology of the coal dust at low and high magnifications, respectively. **(C)** Representative energy-dispersive X-ray spectroscopy layered image and elemental maps showing the spatial distributions of carbon, nitrogen, oxygen, aluminum, silicon, and sulfur. **(D)** Energy-dispersive X-ray spectrum showing the elemental composition of the coal dust. **(E)** Fourier-transform infrared spectrum showing characteristic absorption bands associated with hydroxyl, carbonyl, N–H, Si–O–Si, and Fe–C-related vibrations. **(F)** Intensity-weighted hydrodynamic particle-size distributions of three independently prepared coal dust suspensions measured by dynamic light scattering. Scale bars are indicated in the corresponding panels.

### 3.2 EV-associated material is detected in thyroid cancer cells, and coal dust-stimulated macrophage-derived EV preparations enhance clonogenic growth

To establish an appropriate macrophage–thyroid cancer cell model, EV preparations obtained from coal dust-exposed THP-1 or U937 macrophage-like cells were applied to TPC-1 or ACT-1 thyroid cancer cells (**Figure 2A**). CCK-8 analysis showed a progressive increase in cell viability in all four combinations. The THP-1/TPC-1 combination exhibited the strongest response, with significant differences becoming evident at the later time points, and was therefore selected for subsequent experiments (**Figure 2B**). Colony-formation assays showed that EV preparations derived from coal dust-exposed macrophages increased the clonogenic capacity of TPC-1 cells compared with EV preparations obtained from untreated macrophages. A similar increase was observed after treatment with conditioned medium from coal dust-exposed macrophages compared with the corresponding untreated conditioned medium (**Figures 2C, D**). These findings indicated that soluble and vesicle-associated products released by coal dust-stimulated macrophages could enhance thyroid cancer cell growth under the experimental conditions used. Transmission electron microscopy demonstrated rounded or cup-shaped vesicular structures consistent with small EVs (**Figure 2E**). Nanoparticle tracking analysis showed a relatively narrow particle-size distribution centered at approximately 100 nm (**Figure 2F**). Western blotting detected the EV-associated markers CD9, CD81, and TSG101, whereas the endoplasmic-reticulum protein Calnexin was not detected, supporting enrichment of small EVs in the isolated preparations (**Figure 2G**). Following incubation with TPC-1 cells, PKH26-labeled EV preparations were detected within the cytoplasmic and perinuclear regions. The red fluorescent signals were observed within the boundaries defined by phalloidin-labeled F-actin and around Hoechst 33342-stained nuclei, supporting uptake of labeled EV-associated material by recipient thyroid cancer cells (**Figure 2H**).

**Figure 2:**
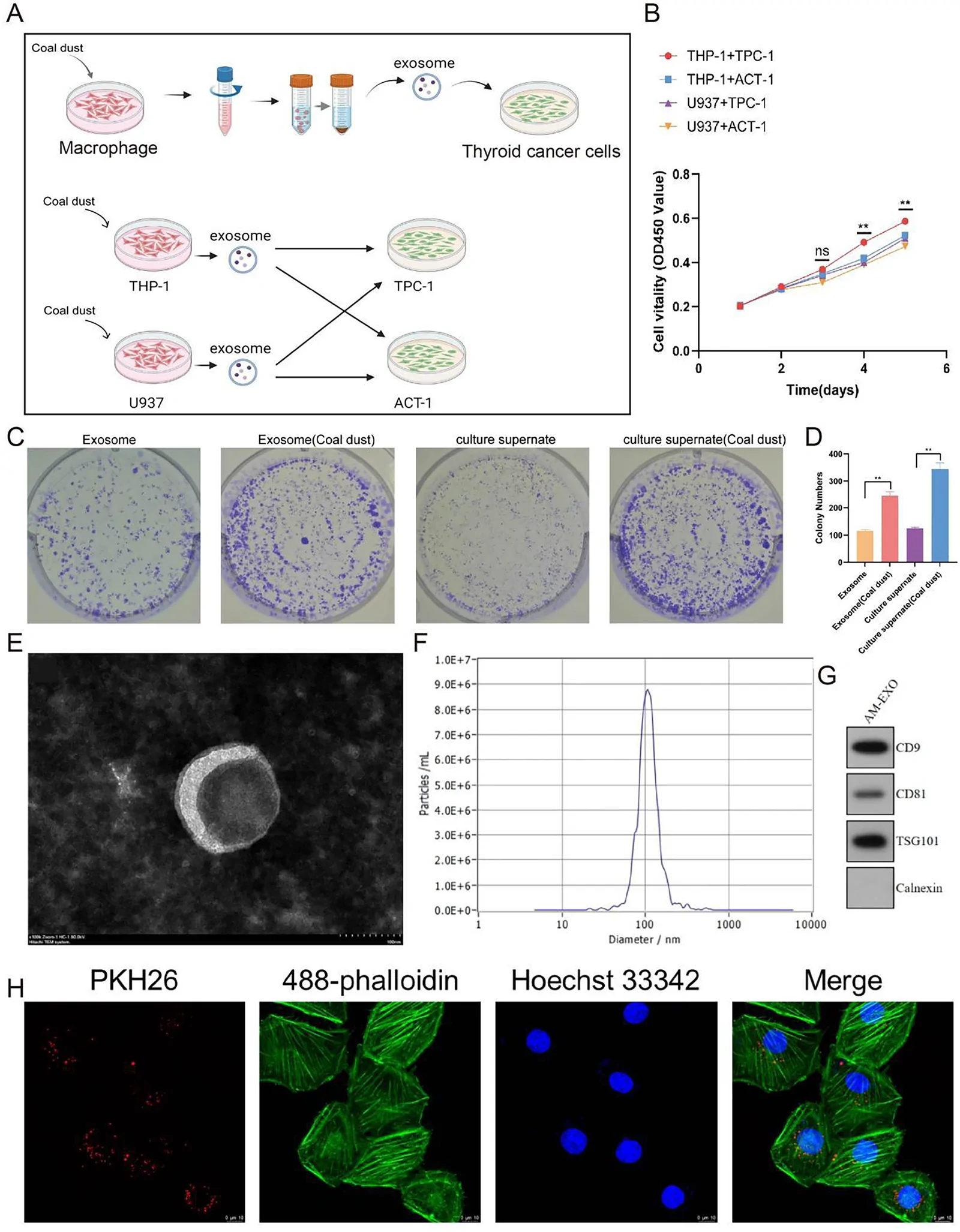
Selection of the macrophage–thyroid cancer cell model and characterization of macrophage-derived EV preparations. **(A)** Schematic illustration of coal dust stimulation, isolation of macrophage-derived EV-enriched preparations, and treatment of thyroid cancer cells, together with the screening strategy involving THP-1 or U937 macrophage-like cells and TPC-1 or ACT-1 thyroid cancer cells. **(B)** Time-dependent viability of TPC-1 and ACT-1 cells following treatment with products derived from coal dust-exposed THP-1 or U937 cells, as determined using the CCK-8 assay. **(C, D)** Representative colony-formation images and corresponding quantification of TPC-1 cells treated with EV preparations or culture supernatants obtained from untreated or coal dust-exposed macrophages. **(E)** Representative transmission electron microscopy image of a macrophage-derived EV preparation. **(F)** Particle-size distribution and concentration of the isolated EV preparation determined by nanoparticle tracking analysis. **(G)** Western blot analysis of the EV-associated markers CD9, CD81, and TSG101 and the negative cellular marker Calnexin. **(H)** Confocal microscopy showing PKH26-labeled EV-associated material in TPC-1 cells. PKH26-associated fluorescence is shown in red, F-actin stained with Alexa Fluor 488-phalloidin is shown in green, and nuclei stained with Hoechst 33342 are shown in blue. Quantitative data are presented as the mean ± SD from three independent experiments. Statistical significance was assessed using two-way ANOVA for the time-course experiment and one-way ANOVA for the colony-formation assay. ns, not significant; **P < 0.01. Scale bars are indicated in the corresponding panels. Legacy “Exo/EXO” labels embedded in original figure panels throughout **Figures 2**–**7** refer to the corresponding EV-enriched preparations and are retained only as legacy graphical group labels; they do not imply demonstrated exosome biogenesis.

### 3.3 miR-205-5p is enriched in coal dust-associated EV preparations and contributes to TPC-1 cell clonogenicity

Small-RNA sequencing revealed distinct miRNA-expression profiles between EV preparations derived from coal dust-exposed macrophages and those derived from untreated controls. Among the differentially expressed miRNAs, miR-205-5p was one of the most prominently increased candidates and was therefore selected for subsequent experimental validation (**Figure 3A**). RT-qPCR showed that the mature miR-205-5p strand was markedly more abundant than miR-205-3p (**Figure 3B**). miR-205-5p was readily detectable in macrophage-derived EV RNA preparations (**Figure 3C**), and its abundance was significantly higher in EV preparations obtained from coal dust-exposed macrophages than in control EV preparations (**Figure 3D**).

**Figure 3:**
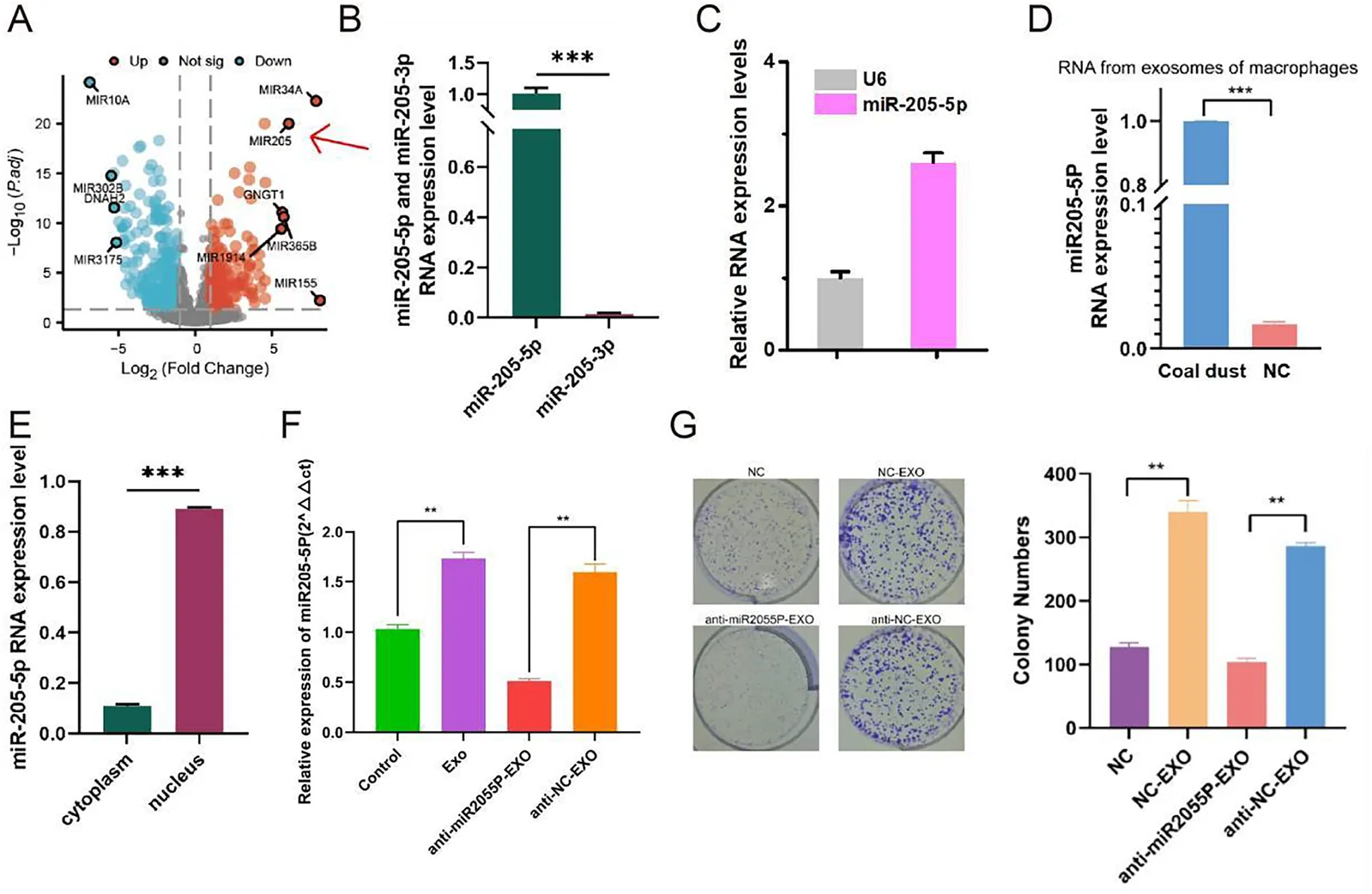
Identification and initial functional validation of EV-associated miR-205-5p. **(A)** Volcano plot showing differentially expressed miRNAs in macrophage-derived EV preparations, with miR-205 highlighted. **(B)** RT-qPCR comparison of the relative abundance of the mature miR-205-5p and miR-205-3p strands. **(C)** RT-qPCR verification of miR-205-5p in macrophage-derived EV RNA, with U6 used as the reference RNA. **(D)** Relative miR-205-5p abundance in EV preparations isolated from coal dust-exposed macrophages and untreated control macrophages. **(E)** Nuclear and cytoplasmic distributions of miR-205-5p in recipient TPC-1 cells following EV incubation. **(F)** Relative miR-205-5p expression in TPC-1 cells subjected to the indicated EV-associated miR-205-5p interventions. **(G)** Representative colony-formation images and corresponding quantification showing the effect of EV-associated miR-205-5p inhibition on the clonogenic capacity of TPC-1 cells. Data are presented as the mean ± SD from three independent experiments. **P < 0.01; ***P < 0.001.

Nuclear–cytoplasmic fractionation of EV-treated TPC-1 cells showed preferential enrichment of miR-205-5p in the nuclear fraction relative to the cytoplasmic fraction (**Figure 3E**). Moreover, incubation with macrophage-derived EV preparations increased intracellular miR-205-5p expression in TPC-1 cells. This increase was markedly reduced when miR-205-5p was inhibited in the EV-producing cells, whereas the corresponding negative-control inhibitor did not produce a comparable reduction (**Figure 3F**). Functionally, EV treatment increased the number of TPC-1 colonies. Inhibition of EV-associated miR-205-5p substantially reduced colony formation, whereas treatment with negative-control inhibitor-containing EV preparations retained a clonogenic response similar to that observed with control EV treatment (**Figure 3G**). These findings identified miR-205-5p as EV-associated cargo enriched following coal dust exposure and implicated it in the EV-associated increase in thyroid cancer cell growth.

### 3.4 EV-associated miR-205-5p reduces apoptosis, while integrated transcriptomic analyses identify CAMK1G as a candidate functional mediator

RT-qPCR confirmed increased miR-205-5p expression in TPC-1 cells following EV exposure and effective suppression of miR-205-5p in the anti-miR-205-5p-EV group (**Figure 4A**). Flow-cytometric analysis showed that EV treatment reduced the proportion of apoptotic TPC-1 cells compared with untreated controls. In contrast, inhibition of EV-associated miR-205-5p increased apoptosis, whereas the negative-control inhibitor EV group did not reproduce this effect (**Figure 4B**). Western blotting showed that EV-treated cells exhibited lower BAX and total caspase-3 abundance than controls, whereas inhibition of miR-205-5p increased both proteins (**Figure 4C**). Because the detected caspase-3 band was approximately 32 kDa, it was interpreted as total caspase-3 rather than cleaved caspase-3; the flow-cytometric assay provided the primary evidence of altered apoptosis. Bulk transcriptomic analysis of EV-treated TPC-1 cells identified enrichment of biological processes related to stress responses, apoptosis, transcriptional regulation, and cell-cycle activity. KEGG analysis highlighted inflammatory pathways, including NF-κB signaling (**Figure 4D**). Differential-expression and pathway analyses prioritized CAMK1G for further experimental investigation (**Figure 4E**). Analysis of the GSE274674 single-cell RNA-sequencing dataset identified major epithelial, immune, and stromal populations within papillary thyroid cancer-related tissues. CAMK1G-associated activity varied among these populations, and CAMK1G-high tumor-cell subsets showed enrichment of inflammatory and stress-associated pathways, including NF-κB-, IL-17-, and TNF-related signaling (**Figure 4F**). Pan-cancer analysis demonstrated heterogeneous CAMK1G expression across tumor types. Within TCGA-THCA, CAMK1G expression was higher in primary thyroid carcinoma tissues than in normal thyroid tissues. Kaplan–Meier analysis did not show a statistically significant overall-survival difference between CAMK1G-high and CAMK1G-low groups (log-rank *P* = 0.36; **Figure 4G**). This TCGA-THCA survival analysis was exploratory and did not support classification of CAMK1G as an independent prognostic marker.

**Figure 4:**
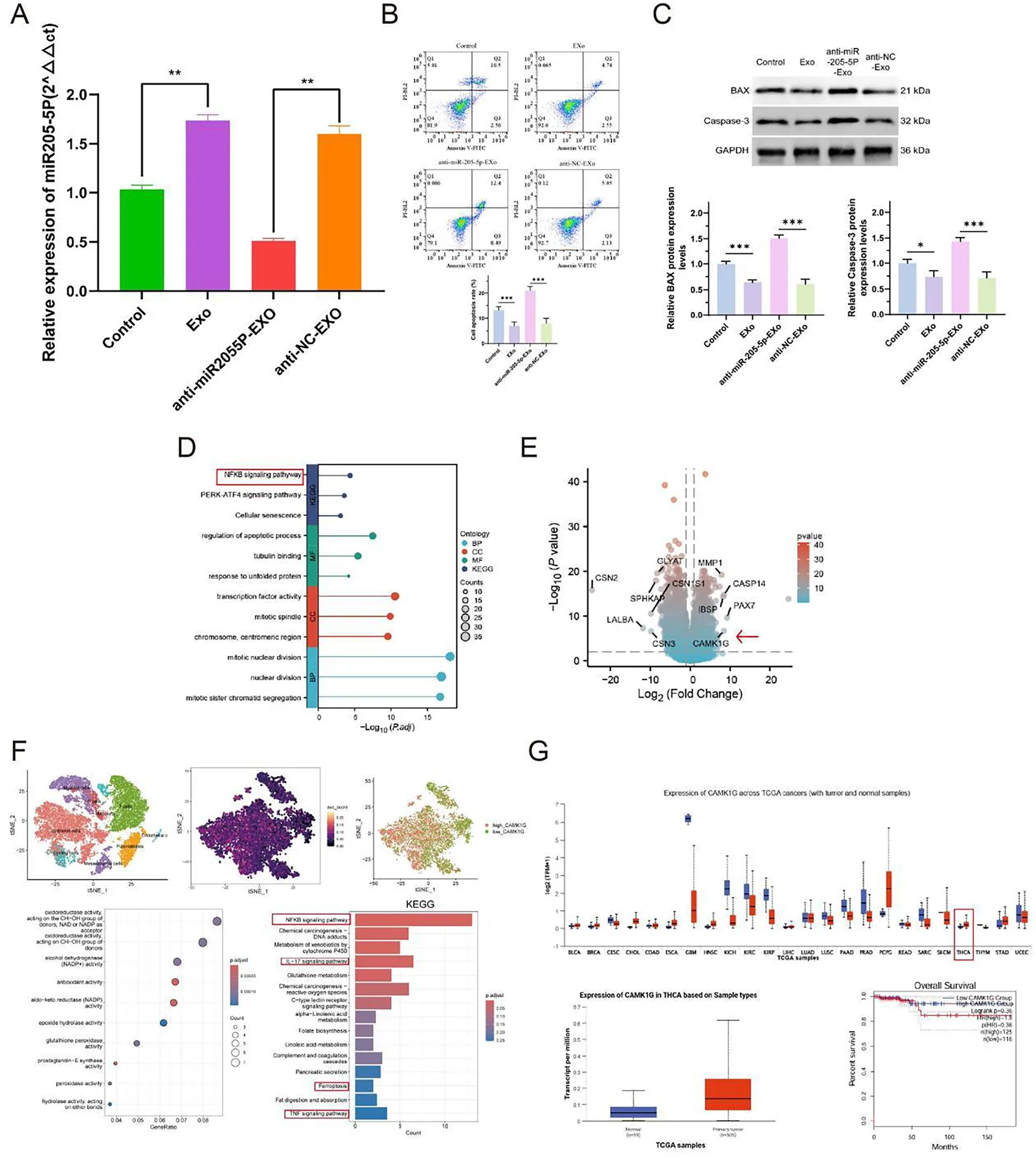
EV-associated miR-205-5p modulates apoptosis in TPC-1 cells, and integrated transcriptomic analyses prioritize CAMK1G as a candidate mediator. **(A)** RT-qPCR analysis of miR-205-5p expression in TPC-1 cells subjected to the indicated EV treatments. **(B)** Representative Annexin V-FITC/PI flow-cytometry plots and quantification of apoptosis in control, EV-treated, anti-miR-205-5p-EV-treated, and anti-NC-EV-treated TPC-1 cells. **(C)** Western blot analysis and densitometric quantification of BAX and total caspase-3 protein expression. GAPDH was used as the loading control. **(D)** Gene Ontology and KEGG enrichment analyses of differentially expressed genes in EV-treated TPC-1 cells. **(E)** Volcano plot of differentially expressed genes, with CAMK1G highlighted. **(F)** Single-cell RNA-sequencing analysis showing major cell populations, CAMK1G-associated AUCell activity, CAMK1G-high and CAMK1G-low cell distributions, and pathway-enrichment results. **(G)** Pan-cancer CAMK1G expression, comparison of CAMK1G expression between TCGA-THCA tumor and normal tissues, and exploratory Kaplan–Meier overall-survival analysis. Quantitative experimental data are presented as the mean ± SD from three independent experiments. *P < 0.05; ***P < 0.001. Adjusted *P* values were used for transcriptomic enrichment analyses.

### 3.5 CAMK1G is functionally associated with the miR-205-5p response, NF-κB-related phosphorylation, and malignant cellular phenotypes

A dual-luciferase reporter assay was performed using constructs containing either the wild-type or mutated miR-205-5p-responsive sequence within the CAMK1G 3′ untranslated region. miR-205-5p reduced the activity of the wild-type reporter, whereas mutation of the predicted response sequence removed this effect (**Figure 5A**). This result supports sequence-dependent repression of the isolated CAMK1G 3′-UTR reporter and does not support a simple direct positive miR-205-5p→CAMK1G regulatory relationship. In contrast, RT-qPCR showed that EV treatment increased both miR-205-5p and endogenous CAMK1G expression in TPC-1 cells, whereas inhibition of EV-associated miR-205-5p reduced endogenous CAMK1G expression and the negative-control inhibitor EV group maintained higher CAMK1G levels (**Figure 5B**). We therefore interpret the reporter result and the endogenous-expression data as mechanistically distinct observations: together they support a functional relationship, but they do not establish the molecular direction or intermediates linking miR-205-5p to endogenous CAMK1G. Western blotting showed that EV treatment increased CAMK1G protein expression and was accompanied by increased phosphorylation of p65 and IκBα without substantial changes in total p65 or total IκBα. Inhibition of EV-associated miR-205-5p reduced CAMK1G expression and attenuated these phosphorylation changes (**Figure 5C**). Two CAMK1G-targeting shRNA constructs reduced CAMK1G expression and were accompanied by lower phosphorylated p65 and phosphorylated IκBα, while total p65 and total IκBα remained relatively stable (**Figure 5D**). These data support functional coupling between CAMK1G abundance and NF-κB-related phosphorylation but do not establish direct phosphorylation of p65, IκBα, or other NF-κB pathway components by CAMK1G.

**Figure 5:**
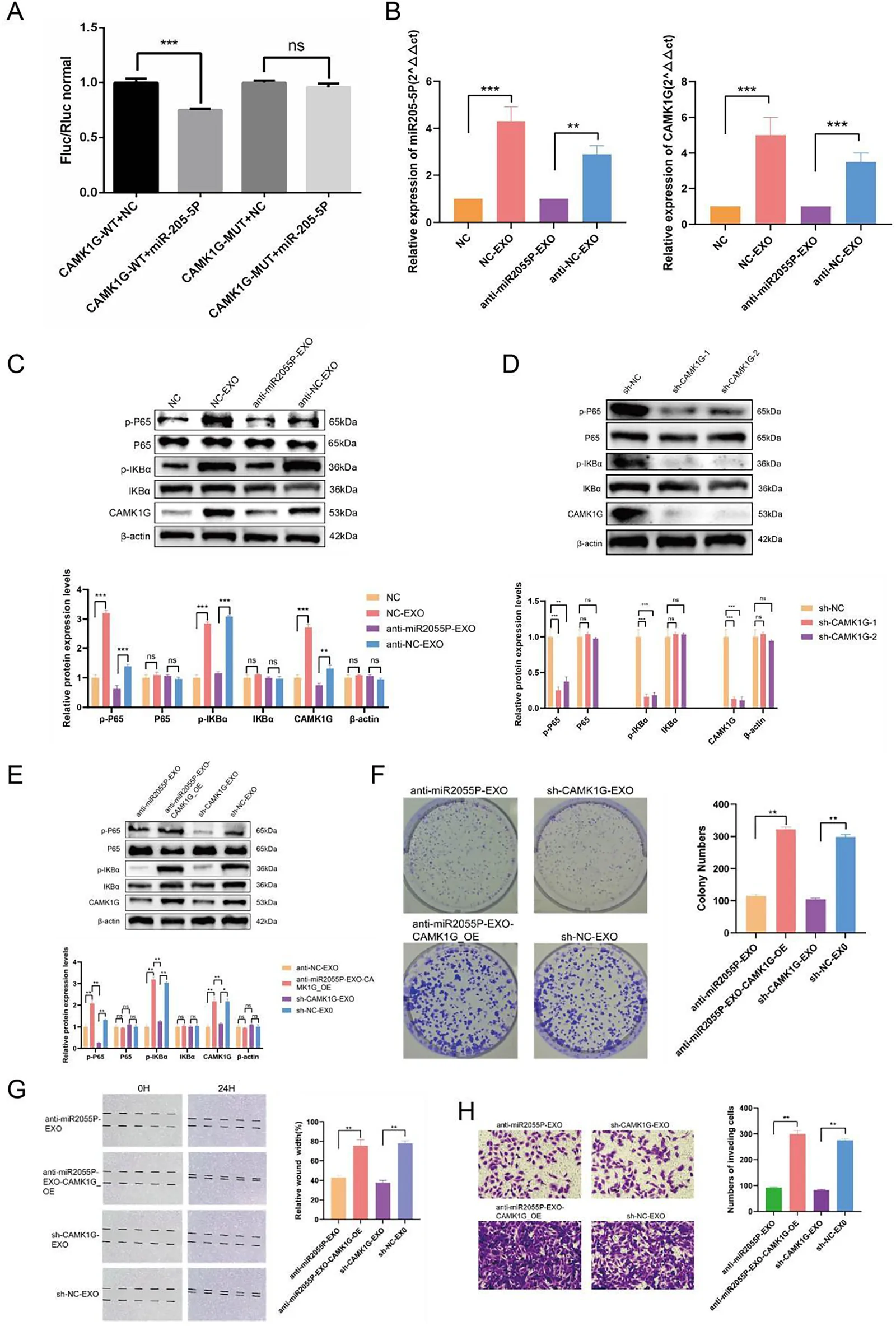
Sequence-dependent interaction between miR-205-5p and the CAMK1G 3′ untranslated region and functional association of CAMK1G with NF-κB-related phosphorylation. **(A)** Dual-luciferase reporter assay in TPC-1 cells co-transfected with miR-205-5p or the corresponding negative control and reporter constructs containing the wild-type or mutated miR-205-5p-responsive sequence within the CAMK1G 3′ untranslated region. **(B)** RT-qPCR analysis of miR-205-5p and CAMK1G expression in TPC-1 cells subjected to the indicated EV treatments. **(C)** Western blot analysis and densitometric quantification of CAMK1G, phosphorylated p65, total p65, phosphorylated IκBα, and total IκBα following inhibition of EV-associated miR-205-5p. **(D)** Western blot validation of CAMK1G knockdown using two independent shRNA constructs. **(E)** Western blot analysis of CAMK1G and NF-κB pathway proteins following miR-205-5p inhibition, CAMK1G knockdown, or CAMK1G restoration. **(F)** Representative colony-formation images and corresponding quantification. **(G)** Representative wound-healing images obtained at 0 and 24 h and quantification of relative wound width. **(H)** Representative Transwell invasion images and quantification of invading cells. Quantitative data are presented as the mean ± SD from three independent experiments. ns, not significant; *P < 0.05; **P < 0.01; ***P < 0.001.

Genetic rescue experiments further examined whether CAMK1G functionally contributes to the cellular effects associated with miR-205-5p. Inhibition of miR-205-5p reduced CAMK1G expression and the phosphorylation of p65 and IκBα. Re-expression of CAMK1G partially restored CAMK1G protein abundance and NF-κB-related phosphorylation, whereas CAMK1G knockdown reproduced the inhibitory effects observed after miR-205-5p suppression (**Figure 5E**). Consistent with these molecular findings, inhibition of miR-205-5p or knockdown of CAMK1G reduced colony formation, whereas CAMK1G re-expression restored clonogenic growth in miR-205-5p-inhibited cells (**Figure 5F**). Wound-healing assays showed reduced closure after miR-205-5p inhibition or CAMK1G knockdown, while CAMK1G restoration increased wound closure (**Figure 5G**). Similarly, Transwell assays showed that CAMK1G restoration partially reversed the reduction in invasive capacity caused by miR-205-5p inhibition, whereas CAMK1G knockdown reduced invasion relative to the corresponding control group (**Figure 5H**). Together, these experiments support CAMK1G as a functional mediator of the observed cellular phenotypes and as functionally coupled to NF-κB-associated activity, while leaving the direct molecular steps between miR-205-5p, CAMK1G, and NF-κB unresolved.

### 3.6 Coal dust exposure induces pulmonary abnormalities, altered thyroid-related serum indices, and increased thyroid CAMK1G expression in mice

Micro-computed tomography performed on day 28 revealed pulmonary abnormalities in coal dust-exposed mice compared with saline-treated controls. In the transverse, coronal, and sagittal planes, coal dust-exposed animals showed increased bilateral pulmonary opacities and structural changes, whereas the control group displayed relatively preserved lung architecture (**Figure 6A**). Serum FT4 levels showed a time-dependent alteration during the 0.5- to 14-day observation period. After an early transient increase, FT4 progressively declined in the exposure group and was significantly lower than that in the control group at later time points (**Figure 6B**). In contrast, serum TPO-Ab concentrations progressively increased following coal dust exposure, with significant differences becoming evident during the later observation period (**Figure 6C**). Immunohistochemical staining showed relatively weak CAMK1G expression in thyroid tissues from control animals. Coal dust-exposed mice exhibited stronger thyroid CAMK1G staining at 1, 2, and 4 weeks, and semiquantitative immunoreactivity scores were higher than those in the corresponding control groups (**Figure 6D**). The 1- and 2-week analyses used the day-7 and day-14 cohorts (n = 5 per group), whereas the 4-week analysis used the separate day-28 cohort (n = 3 per group). These findings showed that experimental coal dust exposure was accompanied by day-28 pulmonary abnormalities, short-term changes in thyroid-related serum indices, and increased thyroid CAMK1G expression.

**Figure 6:**
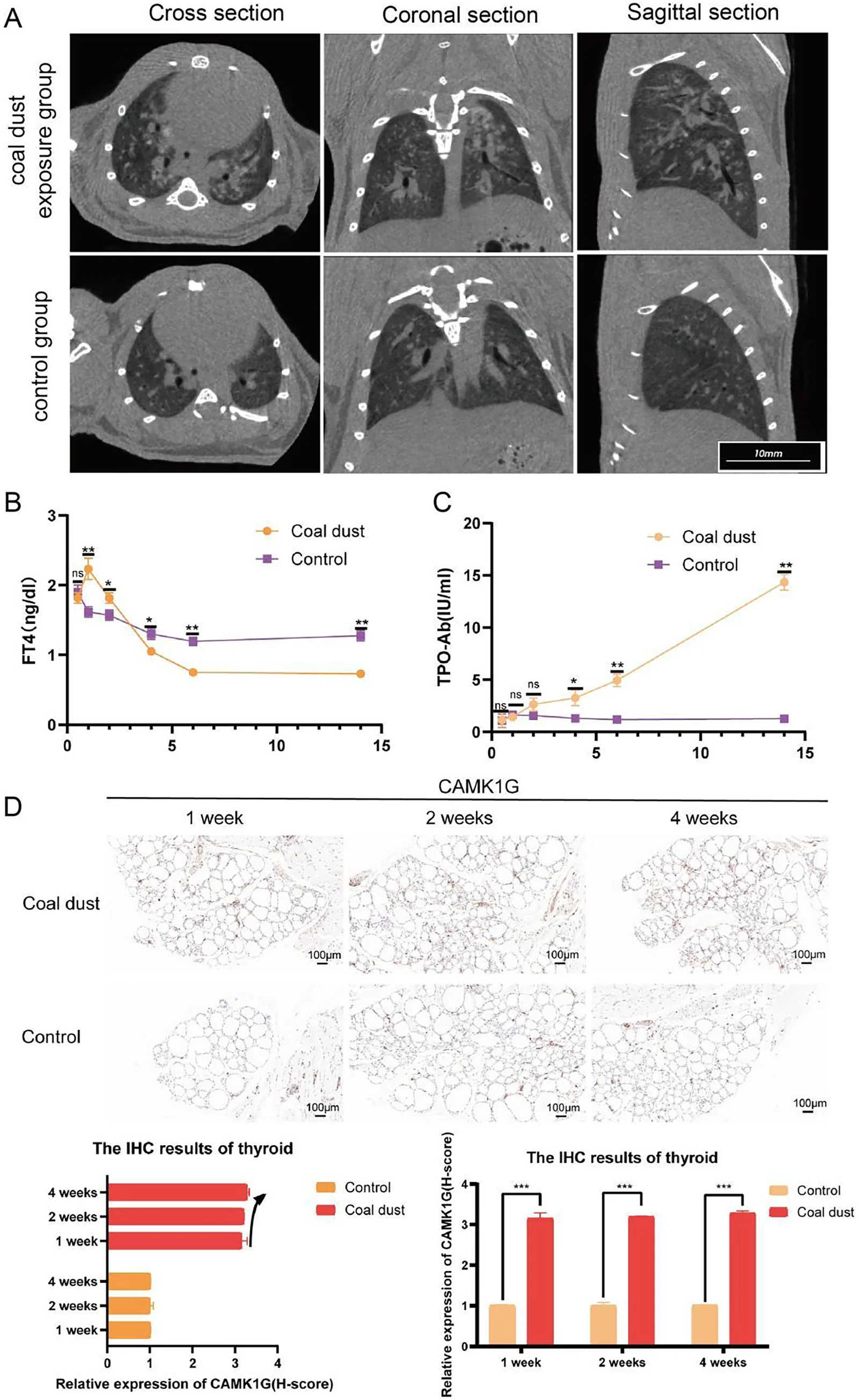
Pulmonary and thyroid alterations in mice following coal dust exposure. **(A)** Representative micro-computed tomography images of the lungs in the transverse, coronal, and sagittal planes obtained on day 28 after the start of exposure (n = 3 per group). **(B)** Temporal changes in serum free thyroxine concentrations at 0.5, 1, 2, 4, 7, and 14 days after the start of exposure. **(C)** Temporal changes in serum thyroid peroxidase antibody concentrations at the same short-term time points. Five mice were analyzed per group at each serum-collection time point. **(D)** Representative CAMK1G immunohistochemical staining of thyroid tissues collected at 1 and 2 weeks from the day-7 and day-14 cohorts, respectively (n = 5 per group), and at 4 weeks (day 28) from the independent day-28 cohort (n = 3 per group), together with semiquantitative immunoreactivity scores. Data are presented as the mean ± SD. Time-course serum data were analyzed using two-way ANOVA followed by Šídák’s multiple-comparison test. ns, not significant; *P < 0.05; **P < 0.01; ***P < 0.001. Scale bars, 100 μm unless otherwise indicated.

### 3.7 Coal dust exposure and macrophage-derived EV preparations promote xenograft growth in PBMC-humanized mice through a CAMK1G-associated mechanism

Flow-cytometric analysis was performed to evaluate human immune-cell engraftment following PBMC transplantation. Human CD45-positive cells were detectable in peripheral blood at day 7 and increased progressively at days 14 and 38. By day 38, representative mice exhibited substantial populations of human CD45-positive leukocytes, accompanied by a corresponding reduction in mouse CD45-positive cells, supporting progressive establishment of the PBMC-humanized model (**Figure 7A**). Successfully humanized mice were implanted subcutaneously with TPC-1 cells expressing control shRNA or CAMK1G-targeting shRNA and were subsequently assigned to vehicle, coal dust, or macrophage-derived EV-preparation treatment. Body weight increased gradually throughout the observation period, with no significant difference between mice bearing sh-NC and sh-CAMK1G tumors (**Figure 7B**). In the sh-NC groups, coal dust exposure and EV-preparation administration resulted in larger tumors than vehicle treatment. Terminal tumor volumes and weights were increased in the sh-NC coal dust and sh-NC EV-preparation groups compared with the sh-NC control group. CAMK1G knockdown reduced tumor burden under each treatment condition and attenuated the increases associated with coal dust exposure or EV-preparation administration (**Figure 7B**). CAMK1G immunoreactivity was markedly reduced in all sh-CAMK1G xenografts, confirming maintenance of the knockdown phenotype *in vivo* (**Figure 7C**). These findings support a functional contribution of CAMK1G to the xenograft-growth response observed after coal dust exposure and experimental administration of macrophage-derived EV preparations.

**Figure 7:**
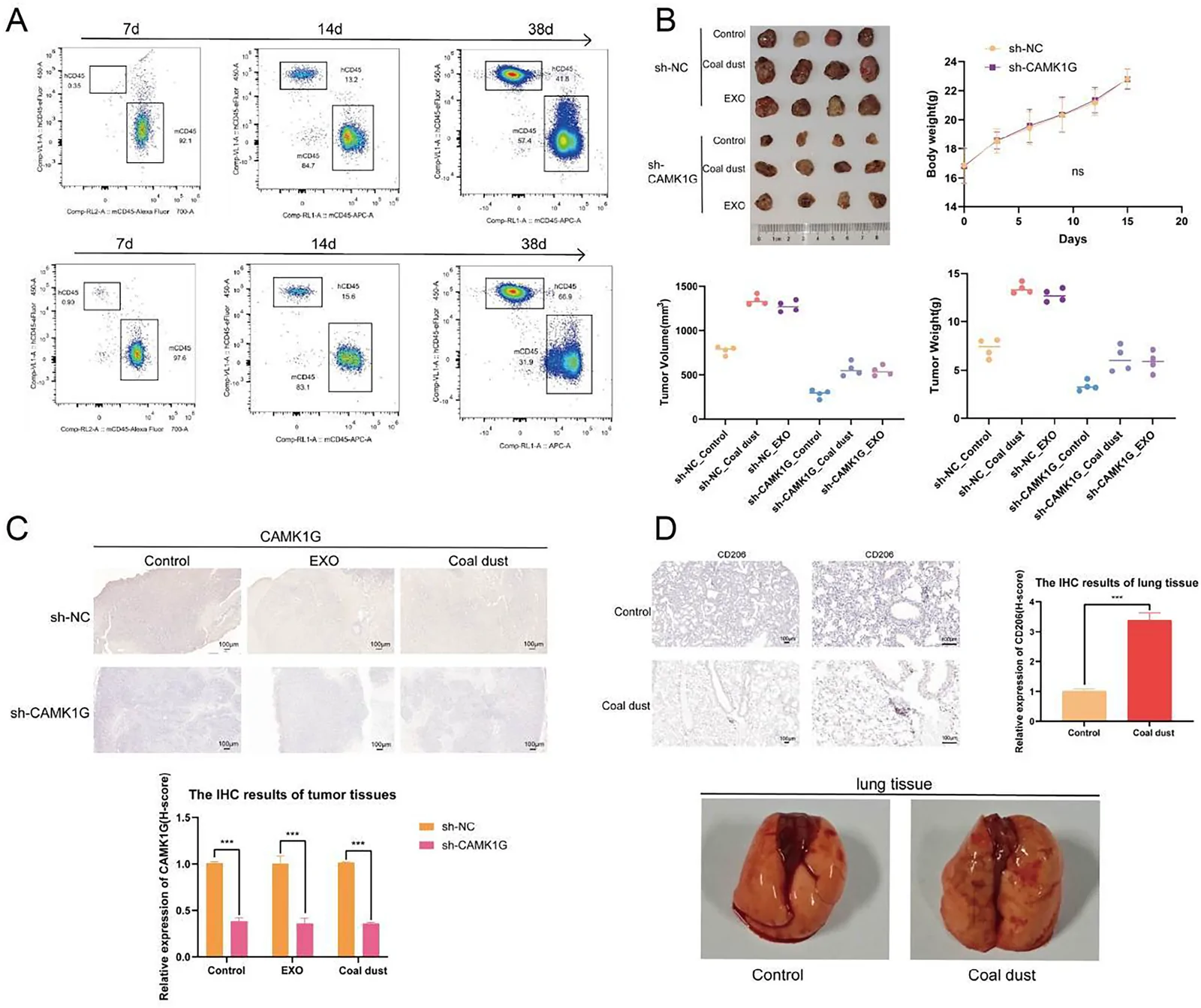
Validation of PBMC-humanized mice and CAMK1G-dependent xenograft responses to coal dust exposure and EV-preparation administration. **(A)** Representative flow-cytometric analysis of human CD45-positive and mouse CD45-positive leukocytes in peripheral blood at 7, 14, and 38 days after PBMC transplantation, showing the temporal development of human immune-cell engraftment. **(B)** Representative excised subcutaneous tumors, body-weight trajectories, terminal tumor volumes, and terminal tumor weights in PBMC-humanized mice bearing sh-NC or sh-CAMK1G TPC-1 xenografts and receiving vehicle, coal dust, or EV-preparation treatment. **(C)** Representative CAMK1G immunohistochemical staining of xenograft tissues and corresponding semiquantitative immunoreactivity scores. **(D)** Representative CD206 immunohistochemical staining and gross morphology of lung tissues from control and coal dust-exposed mice, together with quantification of CD206 immunoreactivity. Four mice were included in each xenograft group. Data are presented as the mean ± SD. ns, not significant; ***P < 0.001. Scale bars, 100 μm. hCD45, human CD45; mCD45, mouse CD45; EXO, legacy graphical label denoting the macrophage-derived EV-enriched preparation.

Lung tissues from coal dust-exposed mice exhibited stronger CD206 immunostaining than control lungs. Semiquantitative analysis confirmed increased pulmonary CD206 immunoreactivity after coal dust exposure (**Figure 7D**). Gross examination also revealed visible pulmonary alterations. These observations were consistent with an increased CD206-positive, M2-like macrophage-associated phenotype but did not by themselves establish comprehensive macrophage polarization. Taken together, the *in vivo* data showed that coal dust exposure and experimentally administered macrophage-derived EV preparations enhanced thyroid cancer xenograft growth, whereas CAMK1G knockdown attenuated these effects. Because endogenous macrophage-derived EVs were not directly tracked from the lung to tumor tissue, the findings provide functional support for the proposed intercellular mechanism but do not establish physiological trafficking of lung-derived EVs to thyroid cancer tissue.

## 4 Discussion

The present study investigated whether extracellular vesicles released by coal dust-stimulated macrophage-like cells influence thyroid cancer cell behavior and examined the potential involvement of miR-205-5p, CAMK1G, and NF-κB-associated signaling. Several principal observations emerged. First, the exposure material exhibited the heterogeneous morphological and compositional features of complex coal dust rather than purified SiO_2_. Second, PKH26-associated material from small extracellular-vesicle-enriched preparations obtained from coal dust-stimulated THP-1 cells was detected within TPC-1 cells, and treatment with these EV preparations was associated with increased clonogenic growth, migration, and invasion and reduced apoptosis. Third, miR-205-5p was enriched in these preparations, and its inhibition attenuated the associated cellular phenotypes. Fourth, transcriptomic analyses and functional experiments prioritized CAMK1G as a candidate mediator linked to NF-κB pathway activity. Finally, coal dust exposure was accompanied by pulmonary and thyroid-related alterations in mice, whereas coal dust exposure or experimental administration of macrophage-derived extracellular vesicles increased xenograft growth in PBMC-humanized mice; these effects were reduced by CAMK1G knockdown. Collectively, these findings support a functional relationship among coal dust stimulation, macrophage-derived extracellular-vesicle signaling, and thyroid cancer progression, but do not establish direct physiological trafficking of endogenous lung-derived vesicles to thyroid or tumor tissue.

Coal mine dust is a complex particulate mixture whose biological activity is determined not only by particle size but also by its carbonaceous fraction, mineral composition, surface properties, and associated contaminants. The SEM, energy-dispersive X-ray spectroscopy, FTIR, and DLS findings in the present study confirmed substantial morphological and compositional heterogeneity. This characterization is important because the effects of complex coal dust cannot be assumed to be equivalent to those of purified crystalline silica. Our previous epidemiological analyses identified an association between occupational coal-mining characteristics and nodular thyroid disease, and subsequent work implicated pulmonary extracellular-vesicle-associated miR-205-5p in CWP-related thyroid abnormalities (12, 13). Nevertheless, those observations were not sufficient to establish a causal relationship between inhaled coal dust and thyroid cancer. The present work extends the earlier findings by examining a defined macrophage-associated signaling mechanism under controlled experimental conditions, while still distinguishing experimental tumor progression from the initiation of human thyroid malignancy.

The concept that inhaled particles may produce effects beyond the lung through extracellular-vesicle-mediated communication is biologically plausible and has received increasing experimental support. Lopez et al. reported that suppression of lung EV production modified neuroinflammatory and behavioral responses after inhalation of particulate matter from a legacy mine site, suggesting that lung-associated circulating vesicles may contribute to systemic toxicological effects (15). More recently, Gao et al. showed that PM_2.5_ exposure increased the release of alveolar macrophage-derived EVs carrying miR-3529-3p, which entered the circulation and promoted vascular endothelial ferroptosis and atherosclerosis; inhibition of EV release reduced the vascular effects (16). These studies support the broader concept that pulmonary particulate exposure can alter EV cargo and influence distant organs. Importantly, however, those studies included EV-release inhibition, circulating-vesicle analysis, or direct evaluation of distal-organ uptake. The present study did not directly trace endogenous lung macrophage-derived EVs into the circulation or thyroid tissue. It therefore provides functional convergence between coal dust exposure and macrophage-derived EV effects but does not provide the same level of evidence for physiological lung-to-thyroid transport.

EVs are heterogeneous lipid-bilayer particles capable of transferring nucleic acids, proteins, lipids, and metabolites between cells, and their cargo can reflect the activation state of the producing cell (17). In the present study, vesicle-like morphology, an approximately 100-nm particle-size distribution, expression of CD9, CD81, and TSG101, and absence of the cellular marker Calnexin supported enrichment of small EVs. However, the combination of ultrafiltration and polymer-based precipitation may also recover heterogeneous EV subtypes and co-isolated non-vesicular material. According to MISEV2023, the term “exosome” should ideally be reserved for vesicles of demonstrated endosomal origin; when biogenesis has not been established, operational terminology such as “small EVs” or “small EV-enriched preparations” is more appropriate (18). Accordingly, the biological effects observed here should be attributed conservatively to the isolated EV-enriched preparations rather than to a completely homogeneous population of endosome-derived vesicles.

The present findings are consistent with growing evidence that macrophage-derived EVs can remodel thyroid cancer cell behavior. Ning et al. demonstrated that EVs derived from M2-like macrophages transferred miR-21-5p to papillary and anaplastic thyroid cancer cells, reduced METTL3 expression, stabilized CD70 transcripts, and contributed to an immunosuppressive microenvironment and resistance to anti-PD-1 therapy (19). Their study established that macrophage-derived EV cargo can directly alter thyroid cancer signaling and used a PBMC-humanized mouse model to assess human immune–tumor interactions. Our results extend this concept to an occupational particulate-exposure setting: rather than examining macrophages conditioned by an established tumor microenvironment, we evaluated macrophage-like cells stimulated by coal dust and identified miR-205-5p and CAMK1G-associated signaling as candidate components of the resulting thyroid cancer cell response. The two studies therefore converge on macrophage EVs as regulators of thyroid cancer biology but differ in the initiating stimulus, molecular cargo, downstream pathway, and biological endpoint. It is also noteworthy that conditioned medium from coal dust-stimulated macrophages enhanced colony formation, indicating that EV-associated cargo might not be the only biologically active component. Cytokines, chemokines, soluble proteins, lipoprotein-associated RNAs, or other extracellular particles may contribute independently or interact with EVs. The isolated EV-enriched fraction nevertheless reproduced part of the conditioned-medium effect, and PKH26-associated fluorescence was detected within recipient TPC-1 cells. These observations support intercellular transfer but should not be interpreted as definitive proof of intact-vesicle internalization because lipophilic dyes can form aggregates or remain associated with non-vesicular lipid structures. Dye-only controls, genetically encoded EV reporters, membrane-protein tagging, or orthogonal imaging approaches would strengthen future uptake studies.

Small-RNA sequencing and RT-qPCR identified miR-205-5p as an EV-associated candidate increased after coal dust stimulation. miR-205-5p is highly context dependent and has been reported to function as either a tumor suppressor or a tumor-promoting regulator according to cell lineage, molecular targets, and microenvironmental conditions (20). This context dependence is especially relevant to thyroid cancer. Li et al. reported that miR-205-5p suppressed papillary thyroid cancer growth and metastasis by targeting GGCT and reducing GGCT–CD44-associated malignant behavior (21). In contrast, the present experiments associated macrophage-derived EV miR-205-5p with increased clonogenicity, migration, and invasion and reduced apoptosis. The difference does not necessarily represent a direct contradiction. Endogenous miR-205-5p expression within thyroid cancer cells, transient delivery of synthetic mimics, and delivery as part of a complex macrophage-derived EV cargo may produce different outcomes. Differences in target availability, miRNA dose, subcellular localization, cell-line background, and accompanying EV proteins or RNAs may also alter the biological response.

Evidence from other cancer systems further illustrates this bidirectional behavior. Tumor-derived exosomal miR-205 was recently shown to promote ovarian cancer progression indirectly by inducing an M2-like macrophage phenotype through PTEN suppression and PI3K/AKT/mTOR activation (22). That study described tumor-to-macrophage communication, whereas the present study examines the opposite direction—macrophage-to-tumor-cell communication. Together, these findings suggest that miR-205-containing EVs may participate in reciprocal communication between malignant epithelial cells and macrophages, although the biological consequence depends strongly on the direction of transfer and cellular context. Our data also extend previous reports in which EV-associated miR-205-5p influenced angiogenesis, cell migration, toxicant-related liver injury, and macrophage responses during lung injury (6–8). However, because EVs contain multiple active cargoes, inhibition of miR-205-5p should be interpreted as demonstrating a contributory role rather than proving that it is the sole mediator.

Nuclear–cytoplasmic fractionation showed preferential enrichment of miR-205-5p in the nuclear fraction of EV-treated TPC-1 cells. Although the canonical function of mature miRNAs is Argonaute-dependent repression of cytoplasmic target transcripts, nuclear miRNAs and Argonaute proteins have been implicated in transcriptional regulation, chromatin-associated processes, RNA processing, and transcript stability (23). Thus, nuclear localization is mechanistically plausible. Nevertheless, nuclear enrichment alone does not demonstrate that miR-205-5p directly activates CAMK1G transcription. This interpretation requires rigorous validation of fraction purity using nuclear and cytoplasmic RNA or protein markers, demonstration of miRNA association with the CAMK1G promoter or nascent transcript, and evidence that transcriptional rather than post-transcriptional processes account for the change in CAMK1G expression.

The relationship between miR-205-5p and CAMK1G is therefore one of the most important findings requiring cautious interpretation. At the endogenous level, EV treatment increased both miR-205-5p and CAMK1G expression, whereas inhibition of miR-205-5p reduced CAMK1G. Furthermore, restoring CAMK1G partially rescued the molecular and cellular effects attenuated by miR-205-5p inhibition. These experiments support a functional positive association between EV-associated miR-205-5p and endogenous CAMK1G expression. In contrast, miR-205-5p reduced the activity of the reporter containing the wild-type CAMK1G 3′-UTR sequence, while mutation of the predicted binding region removed this effect. Thus, the endogenous-expression and reporter results are directionally discordant and cannot be combined to support a simple direct positive miR-205-5p→CAMK1G regulatory claim. The reporter result is consistent with sequence-dependent repression of the isolated 3′-UTR construct. Possible explanations for the endogenous association include indirect regulation through an intermediate repressor, altered transcription or RNA stability, distinct nuclear and cytoplasmic effects, or regulatory elements absent from the reporter construct; however, these possibilities remain hypotheses rather than demonstrated mechanisms. The present findings therefore support a miR-205-5p–CAMK1G functional relationship, but its precise molecular architecture and directionality remain unresolved.

CAMK1G, also known as CLICK-III/CaMKIγ, is a membrane-associated member of the calcium/calmodulin-dependent kinase family (14). Compared with other CaMK family members, its role in malignancy remains poorly defined, and evidence in thyroid cancer is particularly limited. In the current analyses, CAMK1G expression was higher in TCGA thyroid carcinoma tissues than in normal thyroid tissues, and CAMK1G-associated single-cell activity was linked to inflammatory and stress-related pathways. However, overall survival did not differ significantly between the CAMK1G-high and CAMK1G-low groups. Thus, the public-dataset findings support further functional investigation but do not establish CAMK1G as an independent prognostic biomarker. The experimental knockdown and rescue data provide stronger evidence for its functional relevance in the specific cell and xenograft models used here than does the exploratory survival analysis.

The association between CAMK1G and NF-κB activity is biologically relevant because constitutive or stress-induced NF-κB signaling contributes to thyroid cancer cell survival, invasion, inflammatory cytokine production, and treatment resistance (24). Bauerle et al. showed that inhibition of NF-κB reduced primary thyroid tumor growth and experimental metastatic burden and linked NF-κB activity to IL-8 secretion and tumor angiogenesis (25). More broadly, canonical NF-κB activation is regulated through phosphorylation and degradation of IκB proteins, allowing p65-containing complexes to regulate transcriptional programs involved in inflammation and survival (26). Consistent with these studies, EV exposure in the present work increased p65 and IκBα phosphorylation, whereas CAMK1G knockdown attenuated these changes and reduced malignant cellular phenotypes. CAMK1G re-expression partially restored pathway phosphorylation and cell behavior following miR-205-5p inhibition. These intervention and rescue data place CAMK1G functionally upstream of, or coupled to, NF-κB-associated activity in this model, but they do not establish a direct kinase–substrate mechanism. Specifically, the present experiments do not demonstrate that CAMK1G directly phosphorylates p65, IκBα, IKK, or another defined pathway component. Accordingly, we use the terms “CAMK1G-associated NF-κB activity” and “NF-κB-related phosphorylation” rather than “direct activation.” Kinase-substrate assays, kinase-dead CAMK1G rescue, co-immunoprecipitation, phosphoproteomics, and targeted inhibition of candidate intermediates will be needed to define the intervening signaling steps.

The two animal models addressed complementary but distinct questions. In the C57BL/6J exposure model, day-28 micro-computed tomography demonstrated pulmonary abnormalities, while the 0.5- to 14-day serum time course showed changes in FT4 and TPO-Ab and thyroid CAMK1G immunoreactivity was increased at 1, 2, and 4 weeks. Male C57BL/6J mice were selected because the occupational population motivating this work—coal miners—is predominantly male. This choice aligns the exposure model with the principal workforce of interest, but it does not establish sex-independent effects; because female mice were not included, potential sex-specific differences in pulmonary, endocrine, immune, or thyroid responses could not be assessed. The observed endocrine and molecular changes indicate that pulmonary coal dust exposure can coincide with thyroid-related alterations, but reduced FT4 and increased TPO-Ab do not demonstrate thyroid cancer initiation and may reflect systemic inflammation, stress-related endocrine changes, autoimmune-like responses, or altered thyroid hormone synthesis and metabolism. Longer-duration exposure models including both sexes and detailed thyroid histopathology will be required to determine whether these changes evolve into persistent structural thyroid lesions and whether susceptibility differs by sex.

Pulmonary CD206 immunoreactivity also increased after coal dust exposure. CD206 is commonly associated with alternatively activated or M2-like macrophages; however, macrophage activation exists along a dynamic continuum rather than as two fixed M1 and M2 states (27). A single marker cannot fully define macrophage identity or function, particularly in a complex inflammatory tissue. The present finding is therefore best described as increased CD206 expression consistent with an M2-like macrophage-associated phenotype. Confirmation using CD163, ARG1, NOS2, cytokine profiles, multiparameter flow cytometry, or single-cell approaches would provide a more complete assessment of macrophage reprogramming.

The PBMC-humanized xenograft model enabled evaluation of tumor growth in the presence of engrafted human immune cells. Progressive detection of hCD45-positive leukocytes supported successful model establishment before tumor analysis. Similar PBMC-humanized models have been used to examine macrophage EV-mediated immune suppression in thyroid cancer (19). Nevertheless, PBMC-humanized mice have important limitations, including donor-dependent variability, incomplete or short-lived reconstitution of some innate immune populations, xenogeneic immune activation, and the potential development of graft-versus-host disease (28). In the present study, humanization was assessed by hCD45/mCD45 flow cytometry; specific human immune-cell subsets (for example, T cells, B cells, NK cells, and monocytes) and their intratumoral infiltration or functional state were not further phenotyped. Therefore, the contribution of individual human immune compartments to the xenograft phenotype cannot be inferred from the current data. In addition, the subcutaneous TPC-1 xenograft does not reproduce the anatomical, stromal, endocrine, and lymphatic environment of an orthotopic thyroid tumor. Animal numbers were also limited in the longer-term experiments: the day-28 C57BL/6J cohort contained n = 3 mice per group and the PBMC-humanized xenograft groups contained n = 4 mice per group. These sample sizes limit statistical precision and the ability to detect inter-animal heterogeneity. The animal results should therefore be regarded as supportive functional evidence under the experimental conditions rather than definitive modeling of occupationally associated human thyroid cancer, and replication in larger independent cohorts is warranted.

In control-shRNA xenografts, both coal dust exposure and experimentally administered macrophage-derived EVs increased tumor burden, whereas CAMK1G knockdown reduced tumor growth across treatment conditions. This convergence supports the functional importance of CAMK1G. However, tail-vein administration of isolated EVs tests the biological effect of an experimentally delivered dose and bypasses endogenous EV production, pulmonary release, alveolar–vascular transport, circulation, tissue distribution, and clearance. It therefore cannot establish that coal dust-stimulated lung macrophages naturally deliver equivalent quantities of miR-205-5p-containing EVs to thyroid tumors. Future studies should combine macrophage lineage-specific EV labeling, circulating EV immunocapture, tissue biodistribution, and inhibition of pulmonary EV release. Such experiments would permit direct testing of whether endogenous lung-derived EVs mediate the tumor response. The present study has several additional limitations. First, most mechanistic experiments were performed in TPC-1 cells; validation in additional authenticated thyroid cancer cell lines, primary tumor cells, and patient-derived organoids is needed. Normal thyroid follicular epithelial cells were not included, so the present experiments address effects on established thyroid cancer cell phenotypes rather than tumor initiation or selectivity relative to normal thyroid epithelium. Second, although the EV preparations met several commonly used characterization criteria, they were obtained using a precipitation-based method and might contain heterogeneous vesicles and non-vesicular components. Third, the PKH26 uptake experiment lacked the specificity of genetic or protein-based EV-tracing systems. Fourth, the mechanistic basis for the positive association between miR-205-5p and endogenous CAMK1G remains unresolved and is not explained by the inhibitory 3′-UTR reporter result. Fifth, the immediate molecular link between CAMK1G and NF-κB-related phosphorylation was not identified. Sixth, CD206 alone does not establish comprehensive M2 macrophage polarization. Seventh, direct endogenous EV trafficking from the lung to the thyroid or xenograft was not demonstrated. Eighth, endotoxin contamination of the coal dust preparation was not directly quantified; therefore, a contribution from residual endotoxin to macrophage activation cannot be completely excluded despite sterilization of the dust suspension. Ninth, formal blinding was not prospectively documented for the animal experiments. Tenth, the C57BL/6J exposure experiments included only male mice, so sex-dependent responses were not assessed. Eleventh, the day-28 exposure cohort and the PBMC-humanized xenograft groups were small, limiting statistical precision and requiring replication in larger cohorts. Finally, patient-derived blood, thyroid tissue, or circulating macrophage EVs were not examined. A recent systematic review concluded that EVs have potential diagnostic and prognostic value in thyroid cancer but emphasized substantial heterogeneity in isolation, characterization, and reporting and the need for larger prospective validation cohorts (29). Consequently, miR-205-5p and CAMK1G should not yet be proposed as clinical screening or prognostic biomarkers for coal dust-exposed populations.

Overall, this study provides convergent *in vitro* and *in vivo* evidence that EV signaling from coal dust-stimulated macrophage-like cells can influence thyroid cancer phenotypes and that miR-205-5p, CAMK1G, and NF-κB-associated activity participate in this response. The results complement previous studies linking particulate exposure to systemic EV signaling and macrophage-derived EVs to thyroid cancer regulation, while identifying a distinct occupational-exposure-related experimental context. The genetic rescue findings support CAMK1G as a functional mediator, but the miR-205-5p–CAMK1G regulatory mechanism and the direct kinase pathway upstream of NF-κB remain to be established. Direct lineage-specific EV tracing, orthotopic and longer-term exposure models, and validation in samples from coal dust-exposed individuals will be required to determine the physiological and clinical relevance of these findings.

## 5 Conclusion

This study provides evidence that EV-enriched preparations released by coal dust-stimulated macrophage-like cells can influence thyroid cancer cell phenotypes and implicates miR-205-5p, CAMK1G, and NF-κB-associated activity as functionally connected components of this response. EV preparations enriched in miR-205-5p were associated with enhanced clonogenic growth, migration, and invasion and with reduced apoptosis. Endogenous-expression and genetic-rescue experiments supported a functional relationship between miR-205-5p and CAMK1G; however, the dual-luciferase assay showed sequence-dependent repression of the isolated CAMK1G 3′-UTR reporter, so the present data do not establish a simple direct positive miR-205-5p→CAMK1G regulatory mechanism. CAMK1G knockdown reduced p65 and IκBα phosphorylation and malignant cellular phenotypes, whereas CAMK1G restoration partially rescued these effects, supporting CAMK1G-associated NF-κB activity without establishing a direct kinase–substrate link. *In vivo*, coal dust exposure was accompanied by pulmonary abnormalities, increased pulmonary CD206 expression, altered thyroid-related serum indices, and increased thyroid CAMK1G expression; experimental EV administration and coal dust exposure were also associated with increased xenograft growth, whereas CAMK1G knockdown reduced tumor burden. These animal findings are supportive but limited by small group sizes, the use of male-only C57BL/6J exposure cohorts, and the absence of direct endogenous lung-to-thyroid EV tracing. Further studies in larger cohorts of both sexes, with lineage-specific EV tracking and direct mechanistic interrogation of the miR-205-5p–CAMK1G and CAMK1G–NF-κB connections, are required.

## Data Availability

The original contributions presented in the study are included in the article/supplementary material. Further inquiries can be directed to the corresponding authors.
